# The Involvement of Mg^2+^ in Regulation of Cellular and Mitochondrial Functions

**DOI:** 10.1155/2017/6797460

**Published:** 2017-07-05

**Authors:** Ivana Pilchova, Katarina Klacanova, Zuzana Tatarkova, Peter Kaplan, Peter Racay

**Affiliations:** Biomedical Center Martin JFM CU and Department of Medical Biochemistry JFM CU, Jessenius Faculty of Medicine in Martin (JFM CU), Comenius University in Bratislava, Martin, Slovakia

## Abstract

Mg^2+^ is an essential mineral with pleotropic impacts on cellular physiology and functions. It acts as a cofactor of several important enzymes, as a regulator of ion channels such as voltage-dependent Ca^2+^ channels and K^+^ channels and on Ca^2+^-binding proteins. In general, Mg^2+^ is considered as the main intracellular antagonist of Ca^2+^, which is an essential secondary messenger initiating or regulating a great number of cellular functions. This review examines the effects of Mg^2+^ on mitochondrial functions with a particular focus on energy metabolism, mitochondrial Ca^2+^ handling, and apoptosis.

## 1. Impact of Mg^2+^ on Cellular Functions and Intracellular Mg^2+^ Dynamics

Mg^2^^+^ is an essential mineral with pleotropic impacts on cellular physiology and functions [1, 2]. It acts as a cofactor of several important enzymes, especially those requiring ATP in order to be fully functional, such as the various protein kinases, proteins involved in nucleic acid metabolism, or ATPases involved in the transport of various ions [1, 2]. In addition, Mg^2+^ alters the electrophysiological properties of ion channels such as voltage-dependent Ca^2+^ channels and K^+^ channels [3]. The voltage-dependent block of N-methyl-D-aspartate receptor by Mg^2+^ [4, 5] represents an important phenomenon in the neurosciences. Finally, Mg^2+^ can affect the binding affinity of Ca^2+^ to specific Ca^2+^-binding proteins, such as calmodulin [6], S100 [7], troponin C [8], and parvalbumin [9, 10]. The effects of Mg^2+^ on Ca^2+^-handling proteins are responsible for the significant modification of intracellular Ca^2+^ dynamics and signalling [11]. In general, Mg^2+^ is considered as the main intracellular antagonist of Ca^2+^, which is an essential secondary messenger initiating or regulating a great number of cellular functions in various cells [12].

**Figure 1:**
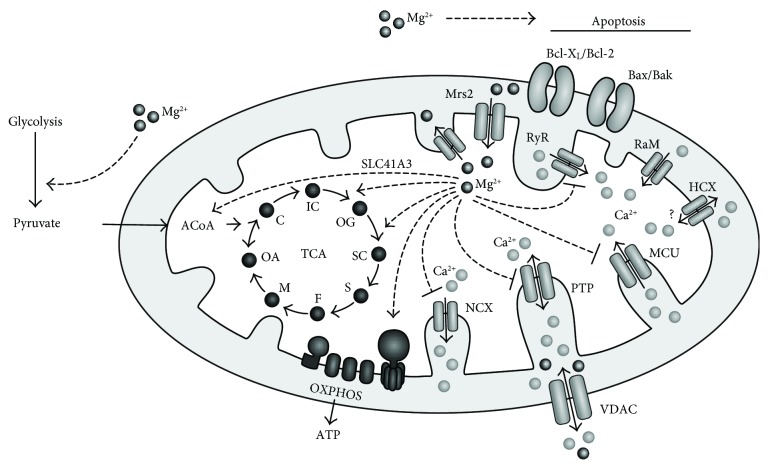
Regulation of mitochondrial functions by Mg^2+^. Mitochondrial Mg^2+^ activates (------>) three dehydrogenases in the mitochondrial matrix: pyruvate dehydrogenase (conversion of mitochondrial pyruvate to acetyl coenzyme A), isocitrate dehydrogenase (conversion of isocitrate to 2-oxoglutarate), and 2-oxoglutarate dehydrogenase (conversion of 2-oxoglutarate to succinyl coenzyme A). In addition, mitochondrial Mg^2+^ activates F_0_/F_1_-ATP synthase, which is the terminal complex of mitochondrial oxidative phosphorylation (OXPHOS). This regulatory activity contributes to mitochondrial energy metabolism. Mitochondrial Mg^2+^ inhibits (------|) Ca^2+^ transporters localised in the inner mitochondrial membrane: Ca^2+^-dependent permeability transition pore (PTP) opening that results in the release of a variety of compounds from mitochondria including Ca^2+^, mitochondrial Ca^2+^ uniporter (MCU), mitochondrial ryanodine receptor (RyR), and mitochondrial Na^+^/Ca^2+^ exchanger (NCX). This regulatory activity contributes to both intracellular and mitochondrial Ca^2+^ homeostasis. Cytoplasmic Mg^2+^ regulates mitochondrial Bax/Bak-dependent apoptosis, which is regulated by proteins of the Bcl-2 family such as Bcl-X_L_, Bcl-2. TCA: tricarboxylic acid cycle/Krebs cycle, ACoA: acetyl coenzyme A, C: citrate, IC: isocitrate, OG: 2-oxoglutarate, SC: succinyl coenzyme A, S: succinate, F: fumarate, M: malate, OA: oxaloacetate, RaM: rapid mode of mitochondrial Ca^2+^ uptake, HCX: mitochondrial H^+^/Ca^2+^ exchanger, SLC41A3: mitochondrial Mg^2+^ efflux system, Mrs2: mitochondrial Mg^2+^ influx channel, VDAC: voltage dependent anion channel.

Recent progress in the field of Mg^2+^ transporter research has led to the identification of plasma membrane Mg^2+^ transporter SLC41A1 [13, 14], mitochondrial Mg^2+^ efflux system SLC41A3 [15], mitochondrial Mg^2+^ influx channel Mrs2 [16], and a mitochondrial Mg^2+^ exporter [17]. Substantial progress has also been achieved with respect to the regulation of whole body Mg^2+^ homeostasis [18]. These discoveries have shed new light on the importance of Mg^2+^ in cellular physiology including mitochondrial functions. Mitochondria have been demonstrated to be capable of both the accumulation of Mg^2+^ and the release of Mg^2+^ [19, 20]. Thus, mitochondria represent an important intracellular Mg^2+^ store. Significant amount of intracellular Mg^2+^ has also been shown to be localised within the lumen of the endoplasmic/sarcoplasmic reticulum (ER/SR) [21]. However, unlike mitochondria, the molecular mechanisms of Mg^2+^ transport through the ER membrane are not yet clear. Since impact of Mg^2+^ on cellular functions was summarised in recent reviews [1–3], we will deal, in this review, with the effects of Mg^2+^ on mitochondrial functions with a particular focus on energy metabolism, mitochondrial Ca^2+^ handling, and apoptosis (Figure 1).

## 2. Impact of Mg^2+^ on Energy (Oxidative) Metabolism

The oxidation of coenzymes (reduced in glycolysis, reaction catalysed by pyruvate dehydrogenase complex, *β* oxidation, and Krebs cycle) in the mitochondrial respiratory chain and the consequent mitochondrial oxidative phosphorylation represent the major pathway of intracellular energy production in the form of ATP for all mammalian cells, except for erythrocytes. A small fraction of ATP is produced in the cytoplasm by the oxidation of glucose in the glycolysis pathway. Many of the glycolytic enzymes (hexokinase, phosphofructokinase, phosphoglycerate kinase, and pyruvate kinase) have previously been shown to be sensitive to Mg^2+^. The most important effect is attributable to the MgATP_2_ complex, which is a cofactor for these enzymes, whereas other chelation forms are inactive or inhibitory [22].

The study of the impact of Mg^2+^ on the enzymes of energy metabolism in mitochondria began several decades ago [23, 24]. The earlier approach, which was focused on the description of the Mg^2+^ effect on isolated mitochondrial enzymes [25, 26], has subsequently been substituted by studies focused on the effect of Mg^2+^ on energy metabolism in isolated vital mitochondria [27–29] or vital cells [30, 31]. Some results obtained by the kinetic analysis of isolated enzymes have also been further analysed in more details by mathematical methods [32, 33]. Mg^2+^ has been documented to enhance the activity of three important mitochondrial dehydrogenases involved in energy metabolism. Whereas activities of isocitrate dehydrogenase (IDH) and 2-oxoglutarate dehydrogenase complex (OGDH) are stimulated directly by the Mg^2+^-isocitrate complex [25] and free Mg^2+^ [34], respectively, the activity of pyruvate dehydrogenase complex (PDH) is stimulated indirectly via the stimulatory effect of Mg^2+^ on pyruvate dehydrogenase phosphatase, which dephosphorylates and thus activates the pyruvate decarboxylase of PDH [35]. OGDH is the rate-limiting enzyme of the Krebs cycle and acts as an important mitochondrial redox sensor [36, 37]. The results obtained by the complex investigation of the impact of Mg^2+^ on ATP synthesis, the mitochondrial transmembrane potential, and respiration indicate that OGDH is the main step of oxidative phosphorylation modulated by Mg^2+^ when 2-oxoglutarate is the oxidisable substrate; with succinate, the ATP synthase is the Mg^2+^-sensitive step [29]. Indeed, Mg^2+^ has been shown to be the activator of ATP synthesis by mitochondrial F_0_/F_1_-ATPase [38, 39].

Taken together, the data suggest that Mg^2+^ has significant impact on the metabolic state, which is mediated by its stimulatory effect on the above-mentioned mitochondrial enzymes. However, the mitochondrial metabolic state seems, in turn, to affect the Mg^2+^ concentration of both the matrix [40] and the cytoplasm [41]. Finally, the effect of Mg^2+^ on energy metabolism partially interferes with the stimulatory effect of Ca^2+^ on energy metabolism and mitochondrial Ca^2+^ transport that are particularly important in excitable cells such as neurones [42, 43] and muscle cells [44]. Increase of extramitochondrial concentration of Mg^2+^ that was not associated with increase of Mg^2+^ concentration in mitochondrial matrix led in the presence of Ca^2+^ to the attenuation of state 3 respiration and stimulation of state 4 respiration [45]. This effect was attributed to the Mg^2+^-dependent inhibition of mitochondrial Ca^2+^ uptake (see further) that resulted in decrease of matrix Ca^2+^ concentration [45].

## 3. Involvement of Mg^2+^ in Regulation of Mitochondrial Ca^2+^ Transport

Mitochondria are important players in intracellular Ca^2+^ homeostasis and signalling [46, 47]. In response to specific signals, mitochondria are capable of both the active accumulation of intracellular Ca^2+^ and the release of Ca^2+^ from mitochondria via different Ca^2+^ transport mechanisms localised on mitochondrial membranes (Figure 1). Thus, they are considered as rapid-uptake slow-release buffers of cytosolic Ca^2+^ [48, 49]. In addition to cell signalling, mitochondrial Ca^2+^ plays an important role with respect to metabolism and cell survival [50, 51]. Several molecular mechanisms control mitochondrial Ca^2+^ transport [52].

The transport of Ca^2+^ through the outer mitochondrial membrane (OMM) is mediated via voltage-dependent anion channel (VDAC) that can be modulated in various ways [52], but little is known about the effect of Mg^2+^ on VDAC-dependent Ca^2+^ transport. An early study had shown that Mg^2+^ did not alter single channel activity but modified single current amplitudes in the lower conductance channel [53].

Active mitochondrial Ca^2+^ uptake is mediated by a specific transporter, namely the mitochondrial Ca^2+^ uniporter (MCU), which transfers Ca^2+^ through the inner mitochondrial membrane (IMM) at the expense of the proton gradient generated by the mitochondrial respiratory chain. The rate of uptake has been described to be proportional to the mitochondrial transmembrane potential [54], but, recently, the exponential dependence of the relative Ca^2+^ transport velocity on the mitochondrial transmembrane potential has received greater support [55, 56]. Another physiologically important question is associated with the low affinity of MCU for Ca^2+^ (apparent K_d_ 20–30 *μ*M at 1 mM Mg^2+^) [57]. The discrepancy between the low Ca^2+^ affinity of the MCU observed in vitro and the high efficiency observed in vivo has been explained on the basis of the microheterogeneity of cytoplasmic Ca^2+^ rising during stimulation. The microdomains of high intracellular Ca^2+^ concentration (10–20 *μ*M) have been suggested to be transiently formed in regions of close proximity to mitochondria and Ca^2+^ channels of the ER or of the plasma membrane [58]. MCU-mediated Ca^2+^ transport in isolated heart, kidney, and liver mitochondria is inhibited in the presence of 1.5 mM Mg^2+^ by approximately 50% in the heart and kidney and by 20% in the liver [59]. Similarly, the inwardly rectifying mitochondrial Ca^2+^ current displaying sensitivity to ruthenium red and selectivity to divalent cations, similar to that of MCU, is reduced by 0.5 mM of cytoplasmic Mg^2+^ concentration to 41% of its conductance in Mg^2+^-free solutions [60]. Moreover, mitochondrial Mg^2+^ loading has been shown to suppress MCU Ca^2+^-uptake rates [61]. The data of experimental studies were used for mathematical modelling of MCU-mediated Ca^2+^ transport suggesting a mixed-type inhibition mechanism for Mg^2+^ inhibition of the MCU function [62]. On the contrary, Mg^2+^ increased the rate of the active and ruthenium-red-sensitive accumulation of Ca^2+^ by isolated rat heart mitochondria [63]. The discrepancy has been attributed to the concentration of Ca^2+^ used for measurements. In the last-mentioned study [63], Ca^2+^ uptake was measured at 25 *μ*M Ca^2+^, thus at a concentration that in the absence of Mg^2+^ is enough to open the permeability transition pore (PTP). Although the rate of Ca^2+^ transport mediated by MCU is inhibited by Mg^2+^, the net accumulation of Ca^2+^ in mitochondria was increased because of the Mg^2+^-mediated prevention of Ca^2+^ leakage from mitochondria via PTP.

Some controversial findings have been reported to be related to the mitochondrial accumulation of Ca^2+^ through IMM via the mitochondrial ryanodine receptor (mRyR). Western blot analysis, immunogold electron microscopy, and the high-affinity binding of [^3^H]-ryanodine indicate that a low level of mRyR is localised within IMM [64]. Similarly to MCU, mRyR is inhibited by low concentrations of ruthenium red (1–5 *μ*M) and by Mg^2+^ [64]. However, the IMM localisation of RyRs by immunogold labelling has not been confirmed by another group [65]. Results obtained in our laboratory also argue against the significant physiological importance of mitochondrial Ca^2+^ uptake via mRyR, since only energised rat heart mitochondria are able to accumulate substantial amounts of Ca^2+^ and the accumulation is prevented by the submicromolar concentration of ruthenium red [63]. Finally, the group of Sheu [66] has suggested that, upon Ca^2+^ overload in the matrix, mRyR might be responsible for mitochondrial Ca^2+^ efflux, thus preventing the activation of PTP (see below).

Recent study documented that Mg^2+^ does not affect the rapid mode of mitochondrial Ca^2+^ uptake [67] that represents another mechanism of Ca^2+^ transport through the IMM distinct from MCU [68].

The main route of mitochondrial Ca^2+^ release has previously been demonstrated to depend on the Ca^2+^-induced release of Ca^2+^ from mitochondria (mCICR). The mechanism of mCICR involves the transitory opening of the PTP operating in a low conductance mode. Therefore, Ca^2+^ fluxes from mitochondria are a direct consequence of the mitochondrial depolarisation spike (mDPS) caused by PTP opening [69]. In vitro, both mDPS and mCICR can propagate from one mitochondrion to another, generating travelling depolarisation and Ca^2+^ waves. Mitochondria therefore appear to be excitable organelles capable of generating and conveying electrical and Ca^2+^ signals. In living cells, mDPS/mCICR is triggered by IP_3_-induced Ca^2+^ mobilisation leading to amplification of the Ca^2+^ signals primarily emitted from the ER [69]. As documented in our laboratory, the opening of PTP in the low conductance mode depends significantly on the Mg^2+^ concentration [63]. This is in agreement with the previous study that documented the inhibitory effect of divalent cations including Mg^2+^ on Ca^2+^-dependent opening of PTP [70].

Two additional antiporters are suggested to play an important role with respect to mitochondrial Ca^2+^ release/efflux [51, 57]. In nonexcitable tissues (liver, kidney), such an antiport, appear to be predominantly an H^+^/Ca^2+^ exchanger, whereas in excitable tissues (heart, brain), it appears to be primarily a Na^+^/Ca^2+^ exchanger [71, 72]. The molecule responsible for the Na^+^/Ca^2+^ exchange was identified in 2010 [73]. A possible molecular candidate for the H^+^/Ca^2+^ exchange (Letm1) was reported in 2009 [74], although this proposal is still controversial [75, 76]. As suggested by Takeuchi and coworkers [51], further analysis is necessary to determine whether Letm1 is, indeed, the H^+^/Ca^2+^ exchanger mediating Ca^2+^ extrusion from mitochondria. The transport activity of the Na^+^/Ca^2+^ exchanger is inhibited by Mg^2+^ at concentration 2.5 mM [77], whereas Mg^2+^ does not inhibit the Ca^2+^ flux mediated by the H^+^/Ca^2+^ exchanger Letm1, even at ∼300-fold excess [75].

## 4. Mg^2+^ and Mitochondrial Apoptosis

Mitochondria play an important role in the process of the intrinsic pathway of apoptosis [78, 79]. They are both targets of proteins of the Bcl-2 family that are essential regulators of intrinsic apoptosis pathway initiation [79, 80], and the residence of proteins playing a crucial role in the execution of intrinsic apoptosis (cytochrome c, Smac/Diablo, apoptosis-inducing factor, and endonuclease G) [81]. In some cells, the extrinsic (receptor) pathway of apoptosis is connected to the intrinsic pathway via receptor-initiated cleavage of Bid protein, which is also a member of the Bcl-2 family, and the consequent translocation of truncated Bid (tBid) to the mitochondria [79, 81].

In contrast to the well-established role of Ca^2+^ in apoptosis [82], the role of Mg^2+^ has been largely ignored. Several in vitro studies have suggested the stimulatory role of Mg^2+^ in both the extrinsic and intrinsic pathways of apoptosis. Changes in cytosolic Mg^2+^ concentration have been observed in the glycodeoxycholate-induced apoptosis of hepatocytes [83], during the proanthocyanidin/doxorubicin-induced apoptosis in K562/DOX cells [84] and in the Fas ligand-induced apoptosis of B lymphocytes [85]. The elevation of intracellular Mg^2+^ observed in early phase of apoptosis has been explained by Mg^2+^ being necessary to stimulate the activity of Ca^2+^/Mg^2+^-dependent endonucleases, which are the executors of apoptosis. Patel et al. [83] have shown that the incubation of cells in Mg^2+^-free medium prevents the rise in intracellular Mg^2+^ and reduces nuclear DNA fragmentation. On the contrary, Chien and coworkers [85] have documented that an increase in cytosolic free Mg^2+^ is independent of the extracellular Mg^2+^ concentration and the source of the elevated intracellular Mg^2+^ has been suggested to be in the mitochondria. This suggestion is supported by the discovery of mitochondrial Mg^2+^ efflux and influx transporters [15, 16] and by experiments revealing the efflux of Mg^2+^ from mitochondria with preserved integrity (i. e., high transmembrane potential, no swelling) as the response to the apoptotic compound, gliotoxin [86]. Finally, the upregulation of Mrs2 has been shown to be responsible for the inhibition of the adriamycin-induced apoptosis of a gastric cancer cell line, probably by suppressing Bax-induced cytochrome c release from the mitochondria [87]. On the other hand, recent studies have documented both the elevation of mitochondrial [88] and the decrease of cytoplasmic [89] Mg^2+^ concentrations in some models of the induction of apoptosis.

Previous studies have also documented the impact of Mg^2+^ on cytochrome c release from mitochondria, an event that is followed by apoptosome formation and further progression of mitochondrial apoptosis [79]. Although a promoting effect of Mg^2+^ has been suggested, the impact of Mg^2+^ on cytochrome c release seems to depend on the mechanism of OMM permeability increase. The release of both Bax- [90] and tBid-induced cytochrome c [91] has been shown to be independent of the PTP pore but to be highly stimulated by Mg^2+^. On the contrary, Noxa-induced cytochrome c release is inhibited by Mg^2+^; this can be explained by the ability of Mg^2+^ to inhibit PTP [92], since PTP opening can result in the release of a variety of compounds from the mitochondria including that of cytochrome c leading to apoptosis [81].

## 5. Conclusions

Mitochondrial dysfunction has been implicated in the mechanisms of several serious human pathologies including metabolic [93, 94], cardiovascular [95], and neurodegenerative [96, 97] diseases. As we have discussed above, Mg^2+^ affects mitochondrial functions that have an important impact on cell survival. Recent work on Mrs2 knockdown HeLa cells has unambiguously revealed that the disruption of mitochondrial Mg^2+^ homeostasis has a dramatic impact on a cellular energy status and cell vulnerability [31]. Moreover, mitochondrial extruder SLC41A3 has been shown to be involved in the regulation of the whole-body Mg^2+^ balance [98]. These findings argue for more systematic research in the field of Mg^2+^ and mitochondria. Since mitochondria display significant cell and tissue heterogeneity [49, 99], the impact of mitochondrial Mg^2+^ on cellular physiology can also be anticipated to be cell- and tissue-type-dependent. Experiments on a variety of cell types will be important. In addition, the impact of Mg^2+^ on apoptosis initiation and execution in various cells has to be investigated in more detail. With respect to apoptosis, the cell-type specificity and the cause-consequence relations between apoptosis initiation and changes in the intracellular or mitochondrial concentration of Mg^2+^ are still unclear. Moreover, recent studies strongly point to the importance of ER-mitochondria interactions with respect to mitochondrial functions, Ca^2+^ homeostasis, and dynamics [100, 101]. Since the ER transport of Mg^2+^ is not as clear yet, the study of the transport of Mg^2+^ through the ER membrane and the possible impact of the luminal Mg^2+^ concentration on ER-mitochondria crosstalk and on mitochondrial Mg^2+^ transport and functions will be crucial. Finally, other processes are localised in the mitochondria, which are also considered as the main site of the intracellular production of reactive oxygen species. The effect of Mg^2+^ on these processes has not been discussed in this review, but some interest should be focused on this direction in the future.
